# Cell salvage within adult and pediatric idiopathic scoliosis surgery: A random cross-sectional study

**DOI:** 10.1051/sicotj/2020038

**Published:** 2020-10-08

**Authors:** Omar A. Al-Mohrej, Bayan Al-Torbaq, Raed Hshem, Jason Sayer, Anwar M. Al-Rabiah, Zayed S. Al-Zayed

**Affiliations:** 1 Department of Orthopedic Surgery, King Faisal Specialist Hospital and Research Center 12713 Riyadh Saudi Arabia

**Keywords:** Cell salvage system, Allogenic blood transfusion, Spine surgery, Scoliosis

## Abstract

*Introduction*: This study aimed to evaluate the use of a cell savage and its impact on the amount of allogenic blood transfused to the patients during idiopathic scoliosis surgery. *Methods*: A total of 142 randomly selected patients with scoliosis had been included in this study. The adult group consisted of 78 patients, and the pediatric group, 64 patients. Both groups were divided into subgroups (pre-cell saver era and cell saver era). Data on the following parameters were collected: amount of blood transfused intraoperatively, within 24 h postoperatively, and overall. The number of patients who received transfusion was counted as the number of patients who avoided any transfusion. For statistical purposes, we performed unpaired student *t*-test, chi-square test, and Mann–Whitney test. *Results*: There was no significant difference in adult groups perioperatively. In the pediatric group, there was a statistically significant difference intraoperatively. Economic analysis of blood management showed positive numbers for both groups, where more than 1 unit of blood was transfused. *Conclusions*: Statistical analysis showed the cost-effectiveness of the perioperative use of cell salvage during pediatric scoliosis surgery. Overall, the use of cell salvage during scoliosis surgery had a positive impact on both blood management and patient recovery.

## Introduction

Blood transfusion, while recognized as an essential therapy when needed, is not without risk [1]. The challenges of patient discomfort and suffering represent a cost to healthcare providers such as increased length of stays, increased aftercare, and potential legal costs [2].

Relevant to orthopedics and spine surgery is the evidence that transfusions can increase the risk of morbidity and mortality [3–6]. Weber et al. noted that allogenic transfusion was the only significant predictor of minor wound disturbances after hip replacement surgery [7]. Similar data showed that post-operative infection was reduced when allogenic transfusions were avoided in patients undergoing primary arthroplasty [8]. Al-Mohrej et al. found that surgical interventions for patients with idiopathic scoliosis were generally correlated with intraoperative bleeding that necessitated transfusion, which in turn increased the risk of infection and wound complications [9]. It also costs the hospital in terms of length of stay, pain relief, antibiotic cover, and possibly prolonged home care [10].

Furthermore, the worldwide crisis in blood supply and an increase in the number of procedures that require a high supply of blood as well as more advanced filtration and testing methods, which increase the cost of a unit of blood, warrant the need for other alternatives. As more data are being collected on the value of blood conservation in spine surgery, more demand is being placed on already scarce perfusion resources [11–13]. One method of blood conservation employed in the spine surgery is cell saver [14]. In Saudi Arabia, this situation was addressed in 2011 with the creation of the role of a Blood Conservation Coordinator, whose main role is to manage the cell saver, promote the concept of the service, and train nursing and anesthetic staff to use the device. Cohesion between centers is being developed via a formal training program and broad promotion of the concept.

This paper aims to examine the effects of a reliable cell salvage service on the outcomes of adult and pediatric scoliosis spinal surgery in terms of blood usage and transfusion frequency.

## Material and methods

The data were collected retrospectively from the time cell saver technology was introduced at our hospital to approximately 1 year later as well as from the time before its implementation. As this was a single-center study that focused on a relatively short period, many of the procedures were considered based on merit. Strict inclusion criteria were set: normal preoperative hemoglobin (Hb) level and the same spine levels, operative time, surgical approach, and technique. The curve magnitude was 55+. Patients with non-idiopathic scoliosis were excluded. All of the surgeries were carried out by an adult spine consultant (AMR) and a pediatric spine consultant (ZSZ). Both surgeons met the thresholds for surgical expertise with an experience of performing such surgeries of more than 10 years with a minimum case load of 25 cases per year.

Based on sample size calculations, 142 patients were included: pediatric patients aged ≤16 years and those aged ≥17 years, considered as adults as per the hospital protocol. The number of levels considered for surgery was 13 levels with no pelvis fixation.

In order to keep the interpretation simple and acknowledge the practicalities and limitations of a small cross-sectional study involving randomly selected patients, the variables analyzed were limited to the amount of blood transfused intraoperatively, postoperatively, and overall and how many patients avoided transfusion. Data on the following parameters were collected: amount of blood transfused intraoperatively, 24 h postoperatively, and overall. The number of patients who refused transfusion was also considered.

The decision of bowl size was based on several factors such as procedure, weight, preoperative Hb level, availability of blood, and judgment of the blood conservation coordinator. However, once a regular supply of 125-mL and 70-mL bowls was in place, 225-mL bowls were not used as blood processed from this size was not ready on time for the procedure, and blood may have been transfused at that point. To the best of our knowledge, this is the first paper to consider bowl size as a factor influencing red cell recovery. Haemonetics Corps Cell Saver 5 and 5+ were used to process the blood. Swab washing was also carried out, which improved the yield to the patient by about a third of the entire volume [15].

All statistical analyses were performed using the Statistical Package for Social Sciences (IBM SPSS Statistics for Mac version 22.0. Armonk, NY: IBM Corp). Categorical variables were presented as frequencies and percentages, whereas continuous variables were summarized as the mean ± standard deviation or medians and centiles when the distributions were skewed. To shed light on different aspects of the study, unpaired student *t*-test, chi-square test, and Mann–Whitney test were used. *P* at 0.05 was considered significant for all tests.

All the data in this study involving human participants were collected in accordance with the ethical standards of our institutional research committee and the 1964 Declaration of Helsinki and its later amendments or comparable ethical standards.

## Results

### Adult patient group

The data for group A were taken randomly from the pre-cell saver era, and data for group B, 1 year after the start of the cell saver era. Mean blood use was generally higher in group A than in group B, with a mean difference of −0.128. At a *P* value of 0.606, this was not found to be significant. The median was the same in both groups at 0 units, while the range was 0–3 in group A and 0–4 in group B, none of which were significant at a *P* value of 0.988. No significant difference was found in transfusion avoidance between group A (79.48%) and group B (82.05%).

Postoperatively, no significant results were obtained. There was a mean difference of −0.05 between the groups, at a *P* value of 0.810. The median was 0 for both groups and the range was 0–4; there was no significant difference at a *P* value of 0.845. Regarding patients avoiding transfusion, a *P* value of 1 showed no significance despite the avoidance of 79.48% and 82.05% in group A and B, respectively.

In all, there was a lack of statistical significance; the average blood use was −0.180, indicating higher blood use in group A than in group B, but at *P* value of 0.602, the difference was not significant. The median was slightly higher in group A at one than group B, which was 0. The range in both groups was six units of blood, which was not significant at a *P* value of 0.41 (Table 1).

**Table 1:** Descriptive statistics of adult patients in terms of unit usage and transfusion avoidance.

Descriptive statistics	Peri-operative timing	Group A	Group B	*P*-value
Mean (units)	Intra-operative	0.795	0.667	0.606
	Post-operative	0.436	0.385	0.81
	Total	1.23	1.05	0.602
Range (units)	Intra-operative	0–3	0–4	0.988
	Post-operative	0–4	0–4	0.845
	Total	0–6	0–6	0.41
Median (units)	Intra-operative	0	0	–
	Post-operative	0	0	–
	Total	1	0	–
Avoided transfusion *n*(%)	Intra-operative	23 (58.97)	27 (69.23)	0.479
	Post-operative	31 (79.48)	32 (82.05)	0.858
	Total	18 (46.15)	23 (58.97)	0.269

**Figure 1:**
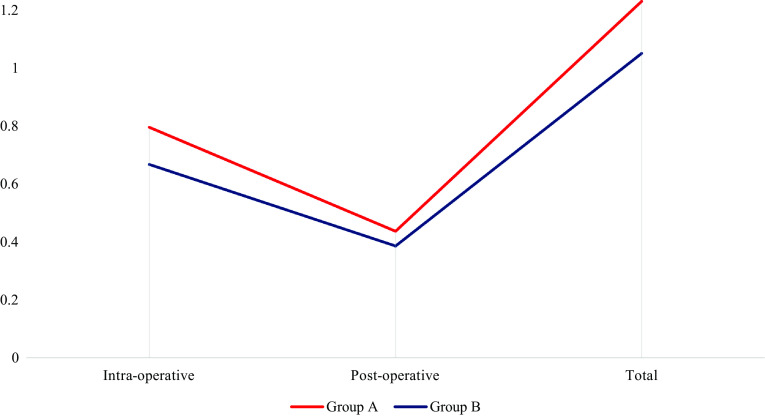
Average blood use in adult scoliosis surgery.

**Figure 2:**
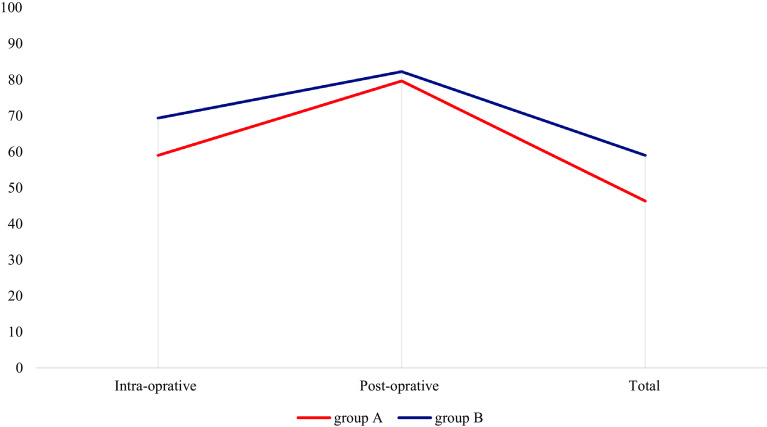
Percentage of patients who avoided transfusion.

The cell saver contributed 30.21 of the 56.21 units transfused, which was 53.74% of the blood transfused intraoperatively. In all, 30.21 units of cell saved blood out of 71.21 units were administered, representing 42.42% of the total blood administered. Figure 1 shows the average blood use in adult spinal surgery, while Figure 2 indicates the percentage of patients who avoided transfusion.

### Pediatric age group

Intraoperatively, a mean of 1.28 units in group C and 0.438 in group D were used, giving a mean difference of 0.843 and *P* value of 0.005. Group C had a higher median (of 1) than group D (median of 0). The range was 0–3 in group C and 0–2 in group D, at a *P* value of 0.016 (Table 2).

**Table 2:** Descriptive statistics of pediatric patients in terms of unit usage and transfusion avoidance.

Descriptive statistics	Peri-operative timing	Group A	Group B	*P*-value
Mean (units)	Intra-operative	1.281	0.438	<0.001
	Post-operative	0.438	0.125	0.037
	Total	1.71	0.59	<0.001
Range (units)	Intra-operative	0–3	0–2	0.016
	Post-operative	0–3	0–1	0.999
	Total	0–5	0–2	0.001
Median (units)	Intra-operative	1	0	–
	Post-operative	0	0	–
	Total	2	0	–
Avoided transfusion *n*(%)	Intra-operative	8 (25.0)	22 (68.8)	0.002
	Post-operative	22 (68.8)	28 (87.5)	0.129
	Total	5 (15.6)	18 (56.3)	0.001

Regarding transfusion avoidance, eight patients avoided transfusion in group C and 22 patients in group D. At a *P* value of 0.002, there was a significant difference between the two groups in favor of the cell salvage group.

A mean of 0.663 units and 0.64 units was found in group C and group D, respectively, with a *P* value of 0.037. This was statistically significant, meaning that less blood was used in the post-operative phase. Mean blood use was 1.719 in group C and 0.594 in group D; there was a mean difference of −1.125 in favor of group D. At a *P* value of <0.001, less blood was used in the cell saver group than in the non-cell saver group. With a median of 2 units, blood use was higher in group C than in group D; the range was also high in the former, but at 0–5 and 0–2, respectively, there was no significant difference (*P* value of 0.302).

**Figure 3:**
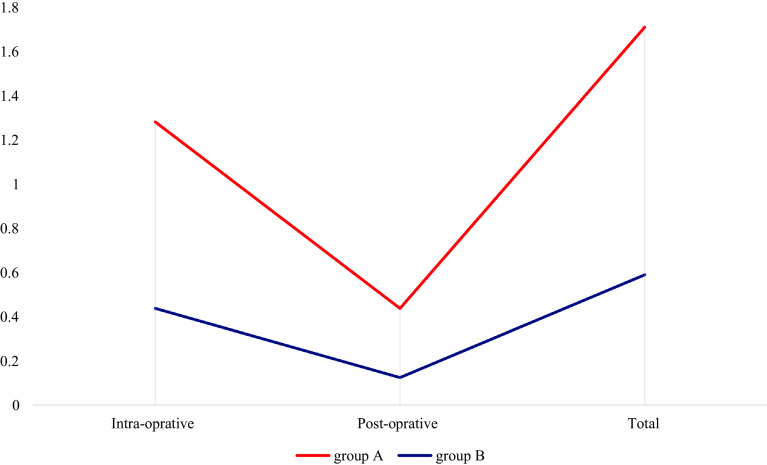
Mean blood transfusion in pediatric groups.

**Figure 4:**
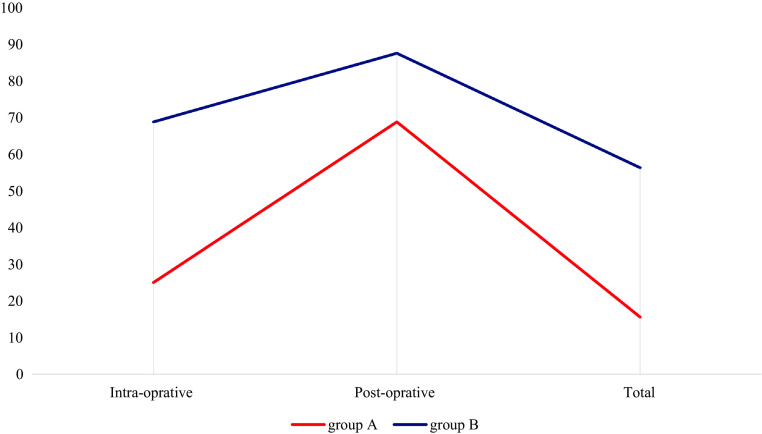
Percentage of pediatric patients who avoided transfusion.

There was a significant decrease in the number of patients who avoided transfusions: 5 in group C and 18 in group D at a *P* value of 0.001. Red cells from the saver contributed to 23.48 units of the blood administered of 37.48 units (62.64%) of the blood transfused. In all, 56.6% of the blood was obtained from the cell saver. Figures 3 and 4 show the mean blood transfusion and the percentage of patients avoiding transfusion in the pediatric age groups.

## Discussion

KFSH&RC is one of the first centers in the Middle East to use the cell saver technology. The hospital is known to undertake surgeries that require a significant amount of blood, such as scoliosis correction surgery. Since its establishment, cell saver was used in 548 patients, most of whom were patients followed by our spine unit at KFSH&RC.

This study presents a snapshot of patients who underwent scoliosis correction surgery to determine the value and cost-effectiveness of cell saver in Saudi Arabia. Although there was no statistically significant change in the adult group, there was a reduction in group B. This lack of statistical significant difference has been noted by several authors in the field of adult spinal surgery [16–18]. However, it can be argued that if cell saver was used before the surgery, the use of allogenic blood may have reduced in group A, but this point remains unknown. A likely explanation for the lack of significance is that the washing of swabs was not fully implemented, which resulted in reduced recovery of red cells. This could have made a positive difference in favor of the cell saver. As per the current data, there was a reduction in blood use and an increase in the percentage of patients who avoided transfusion.

While the evidence was not statistically significant, it showed an improvement in the adult group but not in the pediatric group [9]. From the data and subjective opinions, this type of surgery has had the highest success rate within the blood conservation program at this hospital, and the implementation of swab washing and use of appropriate bowl sizes made a huge contribution to the reduction in the units of blood used and in avoiding transfusion.

Overall, cell salvage in the pediatric group was found to have significant benefits not only in its direct affect but also in its knock-on influence on the total amount of blood used and in assisting patients to avoid transfusion completely. Even in the data showing no statistical difference, there was a reduction in the values of the cell saver group. The majority of the patients were females, as expected for such a pathology. Transfusions in females can lead to issues later on in terms of blood crossmatching as the blood would need to be specific to the patient’s blood group.

In other words, 41 units were used intraoperatively in pediatric group C compared to just 14 in group D, and in all, 55 units were transfused in the non-cell saver group, as compared to 18 in the cell saver group. About 25% avoided transfusion intraoperatively in group C compared to 68.75% in group D; this is an impressive achievement of transfusion avoidance post-operatively of 15.62% in group C and of 56.25% in group D. This reduction is invaluable in terms of cost, resulting in a potential saving of $4552.53 for this surgery group alone in blood transfusion costs. The financial saving resulting from reduction in transfusion-related injury to the patient is incalculable in terms of their well-being as well as bed stay and additional costs to the hospital.

Contrary to earlier studies such as the one by Weiss et al. [18], the sample for pediatric spinal surgery showed a significant, positive change when cell salvage was incorporated into the surgical regime. This finding was in line with that of previous studies on young adults [19, 20], who fall between our two sample types. This finding could be attributed to the technique itself and the implementation of appropriate bowl sizes and swab washing.

Our results have major implications for the patient’s health within the hospital in terms of reduction in transfusion-related reactions, infection, morbidity, and mortality. This also led to a low risk of complications in future life in terms of crossmatch time and intention to have a family. For the hospital, blood is saved and can be used for other patients.

Cell salvage in adults was found to have no benefit in some studies [16, 21, 22]. Our study did not show any statistical benefit, but there was a slight decrease in blood usage and a 25.24% increase in the number of patients who avoided transfusion. These findings and the encouragement of the blood bank that was wondering why blood was being returned to them, show the positive effect of cell salvage. Swab washing ensures that there is efficient use of lost blood, which was evidenced in our findings.

Interestingly, less was blood was used in the postoperative phase in the pediatric groups than in the adult groups. This is an attention-grabbing finding as cell salvage plays no part in this phase except that the level of Hb may have been increased in the intraoperative phase to prevent fluid replacement through blood.

The size of the bowl or washing of swabs was not examined, and both are vital factors in improving the volume of red cells. The time when the data were gathered for this paper was a time of flux for the hospital, where different bowl sizes were being utilized to find the optimum size and swab washing was only being introduced.

The main limitation of our study is its cross-sectional design, which might limit the generalizability of the findings to the general populations. This study was carried out in a single center for a study period of over 2 years. There could be a possibility of bias, and as it was a single-center study, retrospective analysis could have led to the issue of changes in techniques and patient population. The study focused on certain predictors of transfusion; however, many other factors might also play a role. Because of the deficiency in prior data concerning the associations, our multiple testing might lead to spurious associations that need further evidence. For instance, blood loss estimates were difficult to ascertain as “minimal only” was stated on some medical records. Estimating Hb differences was also problematic due to the timing of the blood analysis, and in some cases, blood exams were not carried out at all. Therefore, our study aims to bring awareness and encourage further research about the topic.

## Conclusion

When taken as a whole, the use of cell salvage in spinal surgery has been seen in a positive light; it is of great value in pediatric, young adult, and adult surgery. The benefits could be achieved by efficient use of the cell saver, small bowls, and blood recovery techniques introduced by the blood conservation coordinators and carried out by dedicated surgical, nursing, and anesthetic teams.

## Conflicts of interest

The author(s) declared no potential conflicts of interest with respect to the research, authorship, and/or publication of this article.

## Funding

The authors received no financial support for the research, authorship, and/or publication of this article.
